# Occurrence and Characterization of Fungi and Mycotoxins in Contaminated Medicinal Herbs

**DOI:** 10.3390/toxins12010030

**Published:** 2020-01-03

**Authors:** Ling Chen, Weipeng Guo, Yuqing Zheng, Jinzhen Zhou, Tingting Liu, Wei Chen, Daqing Liang, Meiping Zhao, Yudan Zhu, Qingping Wu, Jumei Zhang

**Affiliations:** State Key Laboratory of Applied Microbiology Southern China, Guangdong Provincial Key Laboratory of Microbial Culture Collection and Application, Guangdong Open Laboratory of Applied Microbiology, Guangdong Institute of Microbiology, Guangdong Academy of Sciences, Guangzhou 510070, China; angusy0662@hotmail.com (L.C.); guowp@gdim.cn (W.G.); zhengyuqing_work@hotmail.com (Y.Z.); zhoujz415@hotmail.com (J.Z.); LIUtt05@hotmail.com (T.L.); onlywei321@hotmail.com (W.C.); liangdaqing2019@hotmail.com (D.L.); zhaomeiping1992@hotmail.com (M.Z.); zhuyd19@gmail.com (Y.Z.); wuqp@gdim.cn (Q.W.)

**Keywords:** mycoflora, medicinal herbs, mycotoxin, contamination, aflatoxins, citrinin, mycotoxigenic fungi, multiplex PCR, HPLC-MS/MS

## Abstract

Traditional medicinal herbs are widely used and may be contaminated with mycotoxigenic fungi during cultivation, harvesting, and storage, causing spoilage and mycotoxin production. We evaluated the predominant mycoflora and extent of mycotoxin contaminations in 48 contaminated samples of 13 different medicinal herbs. In total, 70.8% of herbs were slightly contaminated with aflatoxins (<5 μg kg^−1^). *Codonopsis* radix samples contained ochratoxin A (OTA) (360–515 μg kg^−1^), and *Scutellariae* radix samples contained OTA (49–231 μg kg^−1^) and citrinin (15–53 μg kg^−1^). Forty samples (83.3%) contained fungal contamination. Sixty-nine strains were characterized via morphological and molecular identification. The predominant mycoflora comprised four genera, *Aspergillus* spp. (26.1%), *Penicillium* spp. (24.6%), *Rhizopus* spp. (14.5%), and *Trichoderma* spp. (11.6%). Aflatoxins, OTA, and citrinin were detected in 37 cultures by high-performance liquid chromatography-tandem mass spectrometry. Approximately 21.6% of *Aspergillus* and *Penicillium* isolates produced mycotoxins. One *Penicillium polonicum* strain isolated from *Scutellariae* radix synthesized citrinin. Multiplex PCR analysis showed that three *Aspergillus flavus* strains harbored aflatoxin biosynthesis genes. One *Aspergillus flavus* strain isolated from *Amomi* fructus produced AFB_1_ and AFB_2_. To the best of our knowledge, the citrinin production by *Aspergillus chevalieri* and *Penicillium sacculum* was first reported in this study, which poses a potential risk of mycotoxin contamination in medicinal herbs.

## 1. Introduction

Chinese medicinal herbs are an invaluable asset to China, and widely used in medicinal, edible, and health products. With the continuous development of traditional medicinal herbs, the efficacy of medicinal herbs is recognized and valued, and its safety is a subject of concern. There have been many reports about the international trade obstacles of Chinese medicinal herbs in recent years, mainly on the case of excessive levels of extrinsic harmful residues in medicinal herbs including mycotoxins, pesticides, heavy metals, and sulfur dioxide [1,2,3].

Mildew is the primary cause of mycotoxin contamination among medicinal herbs. Medicinal herbs have been produced primarily through the traditional open, small workshop, and scattered planting business models. No uniform standard or efficient supervision method has been reported for processing, storage, and transportation of medicinal herbs. Mismanagement of medicinal herbs during processing, storage, and transportation and their intrinsic factors and external environmental conditions lead to severe spoilage and deterioration, along with mycotoxigenic fungi contamination. The majority of mycotoxin producers can be found in the fungal genera *Aspergillus*, *Penicillium*, *Fusarium*, and *Alternaria* which concomitantly happen to be the most abundant contaminants of food as well as medicinal herbs. The medicinal herbs (e.g., *Notoginseng* radix et rhizoma, *Scutellariae* radix, *Morindae officinalis* radix) are easily contaminated with molds producing mycotoxins such as ochratoxin A (OTA) [4]. Plant seeds (e.g., *Coicis* semen) are rich in starch, protein, and fat, which are highly susceptible to fungal contamination. The molds and mycotoxins contamination occur frequently in processing due to the heavily rainy days in harvest season as well as inappropriate storage and transportation of *Coicis* semen [5]. Mildew infestation in herbal medicines cause large-scale fungal growth and mycotoxin accumulation, thus affecting the quality and efficacy of medicinal herbs. More than 400 types of mycotoxins reportedly pose a risk to human health. Mycotoxins are secondary metabolites primarily produced by different fungal species. The mycotoxins contaminating medicinal herbs commonly include aflatoxins (AFs), OTA, and citrinin (CTN) [6]. Recent studies have found that Chinese herbal medicines can be simultaneously contaminated by various fungi, especially during storage [7]. The synergistic side effects of several mycotoxins on animal health and agricultural product performance are greater than those of individual mycotoxins [8]. For example, when OTA is used alone, the mortality rate is not high; however, it exerts synergistic effects with aflatoxins to increase the harmful effects of aflatoxins [9].

China is a large-scale producer of medicinal plant resources, with large-scale utilization of medicinal herbs [10]. Most medicinal herbs have a gradual and lasting effect. Some diseases require long-term use of medicinal herbs to achieve therapeutic outcomes. Certain medicinal herbs including *Coicis* semen and *Jujubae* fructus, which are used in medicines and food items, have also long been consumed as health foods. The long-term use of traditional medicinal herbs contaminated with mycotoxins may increase the incidence of adverse reactions [11]. Owing to the limited information available on toxigenic molds in medicinal herbs, this study aimed to evaluate the predominant mycoflora and the extent of fungal contamination in 13 different species of medicinal herbs (*Jujubae* fructus, *Amomi* fructus, *Lycii* fructus; *Notoginseng* radix et rhizoma, *Codonopsis* radix, *Scutellariae* radix, *Morindae officinalis* radix, *Polygoni multiflori* radix; *Coicis* semen; *Poria*; *Ganoderma lucidum*, *Tremella fuciformis*, *Lentinus edodes*) in common use from China, and to investigate their potential ability to produce mycotoxins from isolated fungi, such as aflatoxin B_1_ (AFB_1_), aflatoxin B_2_ (AFB_2_), aflatoxin G_1_ (AFG_1_), aflatoxin G_2_ (AFG_2_), CTN, and OTA.

## 2. Results and Discussion

### 2.1. Mycotoxin Detection

The high-performance liquid chromatography with fluorescence detection (HPLC-FLD) method (Figure S1) was optimized for the detection and quantification of six mycotoxins in medicinal herbs. The chromatogram, limit of quantification (LOQ), and limit of detection (LOD) are summarized in Figure S1 and Table S1. LOD values determined herein ranged from 0.012 μg kg^−1^ for AFB_1_ to 1.3 μg kg^−1^ for CTN. Among 48 medicinal herbs, 39 (81.3%) samples were positive for mycotoxins beyond their LODs (Table 1). Eight samples were simultaneously contaminated with two mycotoxins (AFB_1_ and OTA or CIN), and two samples were simultaneously contaminated with four mycotoxins (AFB_1_, AFB_2_, AFG_2_, and OTA). In the case of medicinal plants, official regulations regarding the presence of only aflatoxins and OTA in medicinal herbs are shared globally among pharmacopoeias and national and organizational regulations. In general, the current legal limit for AFB_1_ in medicinal herbs ranges between 2 µg kg^−1^ and 10 μg kg^−1^, while the limit for total aflatoxins (combined AFB_1_, AFB_2_, AFG_1_, and AFG_2_) ranges from 4 µg kg^−1^ to 20 μg kg^−1^, and the limit for OTA rangs from 15 µg kg^−1^ to 80 μg kg^−1^ [10]. Herein, 70.8% (34/48) of medicinal herbs were contaminated with AFB_1_; however their levels were within the permissible limits (5 µg kg^−1^). AFB_1_ and OTA were simultaneously detected in three varieties of *Codonopsis* radix OTA contamination being severe at 360–515 μg kg^−1^. Contaminant concentrations were higher than 1.2–158.7 μg kg^−1^ reported previously for 23 OTA-contaminated mold samples by Yang et al. [12]. OTA has been classified by the International Agency for Research on Cancer (IARC) as a potential carcinogen (group 2B) on the basis of sufficient animal studies [13]. The recommended provisional tolerable weekly intakes (PTWI) for OTA is 100 ng kg^−1^ body weight (bw), corresponding to approximately 14 ng kg^−1^ bw for 1 d [14]. The Scientific Committee for Food has reported a tolerable daily intake (TDI) of 5 ng kg^−1^ bw [15].

Furthermore, AFB_1_ and OTA were simultaneously detected in one of three *Scutellariae* radix samples, OTA and CTN were detected in the other two samples. These OTA contamination levels (49–515 μg kg^−1^) of *Codonopsis* radix and *Scutellariae* radix should not be disregarded. Considering the vulnerability of several medicinal herbs to OTA, we suggest that a maximum permitted level for this mycotoxin is urgently needed. Further studies are required to develop methods to prevent and control OTA contamination in *Codonopsis* radix and *Scutellariae* radix, including the identification of fungi causing OTA accumulation and establishment of detection standards for *Codonopsis* radix and *Scutellariae* radix to minimize human health risks.

### 2.2. Fungal Contamination

The association between fungal species and medicinal herbs remains unclear owing to complex sources of contamination including extrinsic (environmental and geographical) and intrinsic (constituents of each herbal species) factors [16]. Owing to the widespread presence of fungi in nature, medicinal herbs are often susceptible to fungal contamination inadequately treated during production and storage. The presence of fungi in 48 sample of 13 different medicinal herbs (each ≥ 3 batches) were detected, results showed that 83.3% (40/48) were positive for fungi, demonstrating the majority of Chinese medicinal herbs were contaminated with fungi.

**Figure 1:**
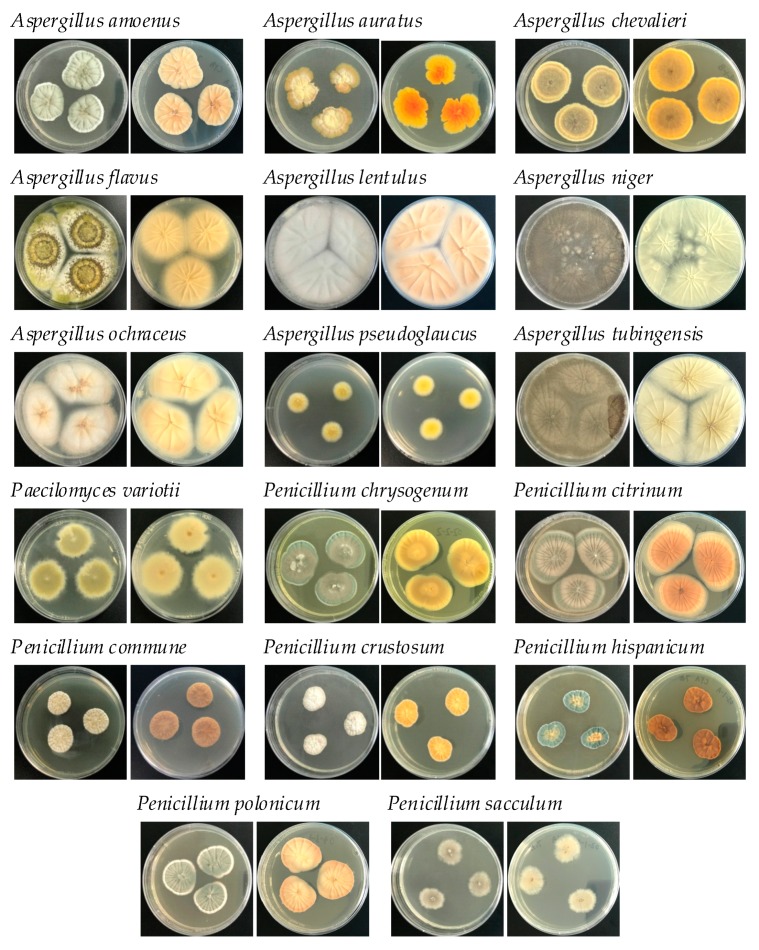
Colony morphology (top and reverse) of *Aspergillus*, *Penicillium*, and *Paecilomyces* species isolated in this study after 3–7 d of incubation at 25 °C in the dark on Czapek Yeast Exatract Agar (CYA).

Sixty-nine fungi (64 strains of mold and 5 strains of yeast) were isolated from 48 medicinal herb samples. Preliminary morphological identification revealed 18 strains of *Aspergillus*, 17 strains of *Penicillium*, 10 strains of *Rhizopus*, 8 strains of *Trichoderma*, 4 strains of *Mucor*, 5 strains of yeast, 2 strains of *Byssochlamys*, 2 strains of *Chaetomium*, 1 strain of *Alternaria*, 1 strain of *Mycosis*, and 1 strain of *Neurospora*. The colony morphology of the primary strains are shown in Figure 1. Traditionally, fungi in medicinal herbs have been divided into two distinct classes: field fungi (e.g., *Fusarium*, *Alternaria*), which invade and produce their toxins before harvest; and storage fungi (e.g., *Aspergillus*, *Penicillium*), which become a problem after harvest [17]. Moreover, some fungi (e.g., *Aspergillus*) might belong to both classes before and after harvest. The predominant mycoflora isolated in this study were storage fungi. Particularly the primary isolated strains from *Codonopsis* radix and *Scutellariae* radix were *Penicillium polonicum*, which potentially produce CTN.

Mildewed medicinal herbs were markedly contaminated with fungi. Overall, *Aspergillus* and *Penicillium* were the most frequent contaminants, concurrent with previous findings [18,19]. Reproduction in *Aspergillus* spp. requires a substrate with a higher water activity, suggesting that the medicinal herbs herein may be not dried in time after harvesting, or that drying was gradual and the duration of dehydration was too long, resulting in subsequent mold propagation. The present drying procedure should be strengthened to reduce the possibility of mold contamination. Furthermore, herbal medicines are often affected with mildew during storage owing to poor management, especially in southern China. Currently, various methods can be used to reduce fungal contamination in medicinal herbs, primarily aiming to prevent, control, and degrade contaminants, i.e., prevention of fungal growth at the source to reduce in contaminated medicinal herbs. Special measures against mildew are required to establish a scientific and reasonable monitoring system, from the beginning of harvesting at the site of origin, with particular attention to the control of mold pollution, thus improving the production conditions of storage, processing, transportation, etc., and preventing subsequent contamination and mold propagation. However, once the mycotoxin is formed or exceeds the standard, subsequent mycotoxin degradation or detoxification methods are required to manage medicinal herbs. Microbial degradation of toxins has recently received increasing attention.

### 2.3. Mycotoxigenic Potentials of the Fungal Isolates

**Figure 2:**
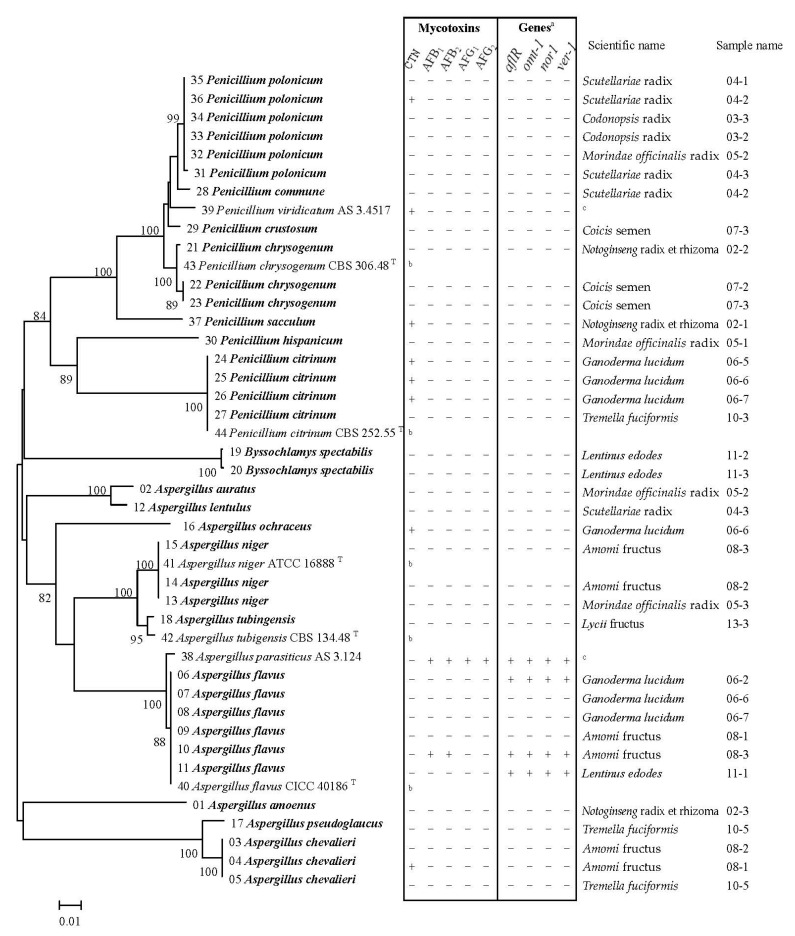
Neighbor-joining tree based on sequence data of ITS and β-tubulin from 44 strains using MEGA 7.0. Bootstrap values are shown in the nodes according to 1000 replications. Only bootstrap values > 80% are shown. Thirty-seven isolates from this study are shown in bold type. “a” aflatoxin biosynthesis genes; “b” untested; “c” standard strain used as positive control; “T” type strain; “+” detected; “−” not detected.

Six mycotoxins (AFB_1_, AFB_2_, AFG_1_, AFG_2_, OTA, and CTN) in 37 strains isolated from 24 samples from 10 different types of medicinal herbs were determined qualitatively by HPLC-MS/MS method (Figure S1). Mycotoxins were detected positive in eight strains (Figure 2), e.g., AFB_1_ and AFB_2_ were simultaneously detected in one strain of *Aspergillus flavus* (from *Amomi* fructus 08-3), CTN was detected in one strain of *Aspergillus chevalieri* (from *Amomi* fructus 08-1), one strain of *Aspergillus ochraceus* (from *Ganoderma lucidum* 06-6), three strains of *Penicillium citrinum* (from *Ganoderma lucidum* 06-5, 06-6, 06-7), one strain of *Penicillium polonicum* (from *Scutellariae* radix 04-2), and one *Penicillium sacculum* (from *Notoginseng* radix et rhizoma 02-1). One strain of *Penicillium polonicum* isolated from *Scutellariae* radix produced CTN after culturing, and CTN was detected in *Scutellariae* radix; however, this is rare. OTA and CTN were detected in *Codonopsis* radix and *Scutellariae* radix; however, we could not isolate the fungi producing these mycotoxins. Medicinal herbs were speculated to be contaminated by mycotoxigenic fungi, and the mycotoxigenic fungi were eliminated in subsequent stages. Due to their wide range of physical and chemical properties, mycotoxins are stable chemical compounds which cannot be destroyed during most processing operations, the mycotoxins still existed in medicinal herbs. The fungi isolated herein were not original contaminated mycotoxigenic fungi. Phylogenetic analysis based on the ITS and β-tubulin gene of the 37 isolates, two reference strains, and other closely related species is showed in Figure 2.

To characterize mycotoxin-producing fungi via multiplex PCR, primers specific for structural and regulatory genes involved in AF biosynthesis (*aflR1*, *omt-1*, *nor1*, and *ver-1*) were assayed in *Aspergillus* and *Penicillium* (Table 2). Consequently, specific fragments amplified from the DNA of isolates with potential mycotoxin-producing abilities. Target genes were not amplified in certain *Aspergillus* strains. The probability of the production of a particular toxin may be predicted in accordance with the presence or absence of an amplification product; however, effective toxin biosynthesis remains unclear even through analytical chemistry analyses [19]. Thirty-nine strains, including 37 isolated from medicinal herbs and 2 standard strains, were assayed for genes involved in AF biosynthesis. Among the 37 strains, 3 displayed positive signals on using gene-based primer sets (Figure 2). Amplification products were obtained from three stains of *Aspergillus flavus*, thus indicating their potential for aflatoxin (AF) production. Only one strain of *Aspergillus flavus* (from *Amomi* fructus 08-3) produced AFB_1_ and AFB_2_ upon analytical chemistry analysis.

AF is a secondary metabolite produced by *Aspergillus flavus* and *Aspergillus parasiticus* and is a derivative of a class of dihydrofuran coumarin. The toxins isolated herein include AFB_1_, AFB_2_, AFG_1_, and AFG_2_, the most toxic one being AFB_1_. In 1993, the International Agency for Research on Cancer reported AFB_1_ as a Class 1 carcinogen [13], since it is 68-fold more toxic than arsenic, second only to botulinum, and is currently the most toxic mycotoxin. The 2015 edition of the Chinese Pharmacopoeia has specified 14 types of medicinal materials including *Nelumbinis* semen, *Myristicae* semen, and *Jujubae* fructus, and five types of animal medicinal materials such as *Hirudo* to detect AFs, and established a permissible limit of 10 µg kg^−1^ in these medicinal material [10]. Approximate 70.8% (34/48) of medicinal herbs in this study were slightly contaminated with aflatoxins (<5 μg/kg). Although 18 strains of *Aspergillus* have been isolated from medicinal herbs, only one culture of *Aspergillus flavus* isolated from *Amomi* fructus produces AFB_1_ and AFB_2_. The reason for this observation may be that *Aspergillus* strains did not harbored aflatoxin biosynthesis genes. Other causes of non-toxicity potentially include environmental factors (temperature, humidity, etc.) or other factors (such as different media). The specific reasons warrant further investigation. *Amomi* fructus may be a suitable substrate for *Aspergillus flavus* produced aflatoxin. More medicinal herbs such as *Amomi* fructus should be included to focus on mycotoxin monitoring.

CTN is a secondary metabolite in filamentous fungi in *Penicillium*, *Aspergillus*, and *Monascus* [22]. *Penicillium* is a toxin-producing genus, primarily including *Penicillium citrinum*, *Penicillium cyclopium*, *Penicillium* *patulum*, *Penicillium urticae*, and *Penicillium viridicatum*. These fungi potentially produce secondary metabolites such as CTN, cyclopiazonic acid, and patulin (PAT). CTN is another mycotoxin that has attracted increasing attention after AF. CTN primarily targets the kidney. CTN exerts cytotoxic and genotoxic effects in both in vivo and in vitro systems. It can cause kidney enlargement, increased urine output, tubular dilatation, and degeneration and necrosis of epithelial cells [23]. However, the molecular mechanisms underlying CTN-mediated biological functions and cytotoxicity remain unknown [24]. CTN and OTA are important mycotoxins, which often coexist in food and feed stuff. Gong [25] assessed the toxicity of OTA and CTN were individually and combinatorially in human embryonic kidney (HEK) 293 cells via the MTT assay and reported synergistic cytotoxic effects upon co-treatment with OTA and CTN, as revealed through significant accumulation of HEK293 cells in the S and G2/M stages. Six strains of *Penicillium polonicum* were isolated, and only one strain from *Scutellariae* radix produced CTN. Three strains of *Penicillium citrinum* were isolated from *Ganoderma lucidum*, and all pure cultures produced CTN. Furthermore, a strain of *Aspergillus ochraceus* isolated from *Ganoderma lucidum* produced CTN. *Ganoderma lucidum* is a traditional Chinese medicinal herb, and polysaccharides are important functional components in the fungus. Over the years, we have performed in-depth studies on *Ganoderma lucidum* and its active substances. The effective development and utilization of *Ganoderma lucidum* resources has broad commercial prospects for human health. Monitoring of molds and mycotoxins has been recommended for *Ganoderma lucidum* to ensure the safety of *Ganoderma lucidum* and *Ganoderma lucidum* products.

CTN was detected in one strain of *Aspergillus chevalieri* isolated from *Amomi* fructus for the first time. Ismail et al. [26] reported that AFB_1_, AFG_1_, and Gliotoxin were produced in the extract of *Eurotium chevalieri var. intermedium* (=*Aspergillus chevalieri var. intermedium*). Among 95 strains of *Eurotium* assayed, CTN was detected in the extract of only one strain of *Eurotium pseudoglaucum* (=*Aspergillus pseudoglaucum*). The melting point of CTN is 175 °C Thus far, few studies have focused on CTN degeneration. Temperature and humidity markedly influence CTN degradation and detoxification. When CTN is heated to 175 °C in a dry environment, it is applied to human cervical cancer cells and does not exert cytotoxic effects. At medium humidity, CTN is degraded and detoxified at 140 °C Therefore, high temperature treatment reduces the CTN content in food, thus reducing CTN intake. Degradation of toxins has recently received increasing attention.

## 3. Conclusions

This study systematically investigated the prevalence of fungi and mycotoxins in medicinal herbs. A relatively large number of medicinal herbs were found to be contaminated, mainly by *Aspergillus* and *Penicillium*. OTA was detected at high levels in *Codonopsis* radix and *Scutellariae* radix. CTN was detected in *Scutellariae* radix at 53 µg kg^−1^. Subsequent analysis indicates that *Penicillium polonicum* was the potential contributors to the high levels of CTN contamination in *Scutellariae* radix. Three strains of *Penicillium citrinum* isolated from *Ganoderma lucidum* can produce CTN. The presence of myco-fungi implies the potential for mycotoxin contamination under suitable conditions, and once the mycotoxins is formed or exceeds the standard, thus could pose a potential health risk to consumers. Considering the toxicological effects of OTA and CTN, a maximum permitted level in several medicinal herbs is needed. Furthermore, these data may help to identify the species of medicinal herbs that are more commonly associated with fungi and mycotoxin contaminations, and to provide useful information for effective prevent, control, and degrade contaminants strategies to ensure the safety of medicinal herbs.

## 4. Materials and Methods

### 4.1. Reagents and Apparatus

All solvents were of high-performance liquid chromatography (HPLC) grade and were purchased from CNW (ANPEL, Shanghai, China). Standards of aflatoxins (AFB_1_: 1.0 mg/L, AFB_2_: 1.0 mg/L, AFG_1_: 0.3 mg/L, AFG_2_: 0.3 mg/L), CTN (>98%) and OTA (>99%) were purchased from Pribolab (Immunos, Singapore) and o2si (Charleston, SC, USA).

HPLC analysis was performed with LC-20AT (Shmadzu Corp., Kyoto, Japan). HPLC-MS/MS analysis was performed with a 1290 Infinity Liquid Chromatograph interfaced to a 6430 Triple Quad Mass Spectrometer system.

MycoSep 226 aflaZon+ multifunctional column were obtained from Romer (Union, MO, USA). Immunoaffinity columns for total aflatoxins (AFB_1_, AFB_2_, AFG_1_, AFG_2_) and Ochratoxin A were obtained from Beacon (Saco, ME, USA). Immunoaffinity columns for citrinin (CitriTest HPLC) were obtained from Vicam (Watertown, MA, USA). Milli-Q water was prepared in our laboratory using the Academic System (Millipore, Molsheim, France).

### 4.2. Sampling

Forty-eight samples of medicinal herbs, comprising 13 different species, were chosen for this study as it is susceptible to fungal growth and mycotoxins production. All samples (Table 3) purchased from the herbal market in China and immediately placed in a sterile polythene bag, properly sealed, and transported to the laboratory and immediately processed to prevent secondary contamination. The medicinal herbs were identified by Diling Chen (Associate professor, Senior postdoctoral researcher, Doctor of Medicine, Guangzhou University of Chinese Medicine, Guangdong Institute of Microbiology, Guangzhou, China) according to Pharmacopoeia of the People’s Republic of China (Edition 2015) [27].

### 4.3. Mycotoxin Analysis

Production of six mycotoxins (AFB_1_, AFB_2_, AFG_1_, AFG_2_, OTA, and CTN) were detected in accordance with the method of Pharmacopoeia of the People’s Republic of China (Edition 2015) [27] and the national food safety standards of China [28,29,30]. The mycotoxins were quantified by comparing peak areas with a calibration curves obtained with standard solutions. Each standard curve comprised five different concentrations of reference solutions, thrice in parallel. All standard curves were linear and R^2^ values were >0.9998. The sensitivity of the method was determined primarily on the basis of the limit of detection (LOD) and limit of quantification (LOQ). The LOD and LOQ of mycotoxins were calculated by considering the average noise signal and adding 3 and 10 standard deviations of noise, respectively. The results are shown in Table S1.

#### 4.3.1. AFB_1_, AFB_2_, AFG_1_, and AFG_2_ Prodution by Medicinal Herbs

Five grams of samples was blended with 20 mL acetonitrile-water (84/16) and extracted using ultrasonography for 20 min. After centrifugation at 6000 rpm for 10 min, the supernatant was purified and concentrated by MycoSep 226 aflaZon+ multifunctional column. 4 mL of the eluate were blow-dried at 50 °C with nitrogen, and the residue was dissolved in 200 μL hexane and 100 μL trifluoroacetic acid, vortex-mixed for 30 s, derived in an incubator at 40 °C ± 1 °C for 15 min. The derivative was blow-dried at 50 °C with nitrogen, and the residue was dissolved in 1 mL of acetonitrile, vortex-mixed for 30 s, passed through a 0.22-μm microporous membrane, and harvested for injection. HPLC analysis was performed with LC-20AT coupled to a FLD detector set to 360 nm excitation and 440 nm emission. The sample was separated using a ZORBAX Eclipse Plus C18 column (250 × 4.6 mm, 5-Micron, Agilent Technologies Co., Ltd., Santa Clara, CA, USA). The mobile phase consisted of (A) water and (B) acetonitrile, at a flow rate of 1.0 mL/min in gradient mode, 0–13 min: 20%–30% of B, 13–17 min: 30%–40% of B, 17–18 min: 40% of B, 18–20 min: 40%–20% of B, 20–22 min: 20% of B. The retention time of the AFB_1_ was about 11.7 min, 20.0 min for AFB_2_, 9.5 min for AFG_1_ and 17.2 min for AFG_1_.

#### 4.3.2. OTA Prodution by Medicinal Herbs

Five grams of samples was blended with 20 mL methanol-water (80/20) and were placed for 30 min in a shaker, then filtered using quantitative filter paper. To 10 mL of extract, 40 mL of phosphate buffer solution (PBS, 8 g NaCl, 1.2 g Na_2_HPO_4_, 0.2 g K_2_HPO_4_, 0.2 g KCl, and brought up to 1 L with distilled water, pH 7.4 ± 0.1) was added, followed by glass fiber filter paper. The filtrate was purified by ochratoxin A immunoaffinity column (Beacon, 3 mL). The immunoaffinity column was rinsed with 10 mL PBS buffer, 10 mL water and 1.5 mL of methanol in turn. The eluate was blow-dried at 45 °C with nitrogen, and the residue was dissolved in 500 μg of mobile phase. HPLC analysis was performed with LC-20AT coupled to a FLD detector set to 333 nm excitation and 460 nm emission. The sample was separated using a ZORBAX SB C18 column (150 × 4.6 mm, 5-Micron, Agilent Technologies Co., Ltd., Santa Clara, CA, USA). The mobile phase was acetonitrile-water-glacial acetic acid (96/102/2), at a flow rate of 1.0 mL/min in isocratic mode. The retention time of the OTA was about 5.5 min.

#### 4.3.3. CTN Prodution by Medicinal Herbs

Five grams of samples was mixed with 25 mL methanol-water (70/30) and homogenated at high speed for 2 min, and then filtered using quantitative filter paper. One milliliters of the filtrate was diluted with 49 mL of phosphoric acid solution (10 mmol/L) and filtered through a glass fiber filter paper. The filtrate was purified by Citrinin Immunoaffinity Column (CitriTest HPLC, 3 mL, VICAM, Milford, MA, USA). The extract was eluted with 1.0 mL methanol–phosphoric acid solution (70/30) at a rate of 1–2 drops/s and then evaporated to dryness under a stream of nitrogen and analyzed using HPLC. HPLC analysis was performed with LC-20AT coupled to a FLD detector set to 350 nm excitation and 500 nm emission. The sample was separated using a ZORBAX SB C18 column (150 × 4.6 mm, 5-Micron, Agilent Technologies Co., Ltd., Santa Clara, CA, USA). The mobile phase consisted of (A) acetonitrile and (B) phosphoric acid solution (10 mmol/L pH 2.5), at a flow rate of 1.0 mL/min in gradient mode, 0–10 min: 20% of A, 11–14 min: 20%–70% of A, 14–18 min: 70%–20% of A, 18–20 min: 20% of A. The retention time of the CTN was about 7.5 min.

### 4.4. Isolation and Purification

Ten grams of samples was added to 90 mL of sterile 0.85% physiological saline and mixed to prepare a 1:10 test solution [27]. Serial decimal dilutions were obtained and cultured on Sabouraud dextrose agar (SDA, 10 g of peptone, 40 g of dextrose, 15 g of agar, 0.1 g chloramphenicol, and disosslved with to 1 L with distilled water) [31] at 25 °C for 5–7 d, and the colony morphology was observed and recorded. For uncut samples, the surface of the sample was directly scraped off with a sterile inoculating loop and inoculated onto SDA or malt extract agar medium (MEA, 130 g of malt extract, 15 g of agar, 0.1 g chloramphenicol, and dissolved with 1 L with distilled water), and the mildewed parts were primarily scraped off. After incubation at 25 °C for 5–7 d, colony morphology was observed and recorded. Colonies with different morphological characteristics were picked and inoculated on SDA, separated, and purified to obtain pure cultures, and colony morphology and microbial growth in the medium were observed.

### 4.5. Phenotypic Characterization

For macromorphological observations, SDA, MEA, and Czapek Yeast Exatract Agar (CYA, 5 g yeast extract, 3 g of NaNO_3_, 1 g of K_2_HPO_4_, 0.5 g KCL, 0.5 g MgSO_4_·7H_2_O, 0.01 g FeSO_4_·7H_2_O, 30 g sucrose, 15 g of agar, and disosslved with 1 L with distilled water) was used. Isolates were inoculated at one or three points on each plate of each medium and incubated at 25 °C in the dark for 3–7 d. For microscopic morphological observations by biological microscope CX21FS1C (Olympus Ltd., Guangzhou, China), microscopic mounts were carried out in lactic acid from colonies cultured on MEA, and a drop of ethanol was added to eliminate air bubbles and excess conidia [8].

### 4.6. DNA Extraction, Sequencing, and Analysis

DNA was extracted from the cells, using the E.Z.N.A.^®^ fungal DNA mini kit (Omega Bio-tek, Norcross, GA, USA) in accordance with the manufacturer’s instructions. Three gene regions of the genomic DNA were amplified and sequenced: (1) the benA-specific primer pair Ben2f/Bt2b [32] was used to amplify the β-tublin gene. (2) The calmodulin gene was amplified using the CF1L/CF4 [33] primer pair. (3) The internally transcribed spacer (ITS) region was amplified using the ITS1/ITS4 primer pair [34]. Sequencing analysis was performed at BGI Co. Ltd. (Guangzhou, China) and the sequencing results were analyzed using BLAST by searching the NCBI nucleotide database for the genus and species of the isolates.

### 4.7. Qualitative Determination of Mycotoxin-Producing Strains

Thirty-seven strains (including 18 strains of *Aspergillus*, 17 strains of *Penicillium*, and 2 strains of *Byssochlamys*) were used to determine the presence of AFB_1_, AFB_2_, AFG_1_, AFG_2_, CTN, and OTA. Among the total isolates obtained from the samples, 37 strains potentially producing six mycotoxins were cultured in 200 mL Sabouraud dextrose medium (SD, 10 g of peptone, 40 g of dextrose, 0.1 g chloramphenicol, and disosslved with 1 L with distilled water) at 25 °C and 200 rpm for 5 d. Furthermore, standard strains *Penicillium viridicatum Westling* AS 3.4517 and *Aspergillus parasiticus Speare* AS 3.124 were used as the positive control. *Penicillium viridicatum* reportedly produces CTN [35]; *Aspergillus parasiticus*, AFB_1_, AFB_2_, AFG_1_, and AFG_2_ [20].

All liquid cultures of strains were sterilized by IRM G65 autoclaves (Frankfurk, Germany). The sterilized liquid cultures were ultrasonically extracted for 30 min, vortex-mixed for 10 min, and then transferred to a 50-mL centrifuge tube, and centrifuged at 3500 rpm for 5 min, and the supernatant was purified using the quantitative filter paper and transferred to another centrifuge tube. To 10 mL of extract, 20 mL of PBS was added, followed by immunoaffinity column chromatography, as previously reported [21]. The flow rate was maintained at 1–3 mL/min. The immunoaffinity column was rinsed with 10 mL PBS buffer and 1 mL of methanol. In three independent samples, and the eluate was blow-dried at 55 °C with nitrogen, and the residue was dissolved in 1 mL of acetonitrile:water (1:1), vortex-mixed for 30 s, passed through a 0.22-μm microporous membrane, and harvested for injection. HPLC-MS/MS analysis was performed with a 1290 Infinity Liquid Chromatograph interfaced to a 6430 Triple Quad Mass Spectrometer system. The sample was separated using a Hypersil Gold C18 column (2.1 × 100 mm, 3-Micron). The mobile phase A: aqueous containing 1% formic acid, mobile phase B: acetonitrile; programmed gradient elution, flow rate: 0.3 mL/min; column temperature: 35 °C; injection volume: 5 μL. The mass spectrometer was operated in the positive mode with multiple reaction monitoring (MRM). Two precursor-to-product ion transitions were simultaneously monitored at *m*/*z* 313.0–285.0, *m*/*z* 313.0–269.0 for AFB_1_; *m*/*z* 315.0–287.1, *m*/*z* 315.0–259.1 for AFB_2_; *m*/*z* 329.1–243.2, *m*/*z* 329.1–311.0 for AFG_1_; *m*/*z* 331.0–313.0, *m*/*z* 331.0–245.0 for AFG_2_; *m*/*z* 404.1–239.0, *m*/*z* 404.1–358.0 for OTA; and *m*/*z* 251.0–233.0, *m*/*z* 251.0–205.0 for CTN (Figure S2).

### 4.8. Multiplex PCR

To optimize the multiplex PCR assay to detect AF-producing fungal species in samples of medicinal herbs on the basis of genes [36,37] involved in mycotoxin biosynthesis (Table 2), reference fungal strains were confirmed via monoplex PCR. The multiplex PCR was standardized by empirically varying critical factors affect multiplexing, such as primer concentrations, template concentration, and annealing temperature. The PCR conditions were as follows: initial heat activation of DNA polymerase at 94 °C for 2 min, followed by 35 cycles of denaturation at 94 °C for 30 s, annealing at 55 °C for 30 s, extension at 72 °C for 90 s, and a final extension at 72 °C for 7 min. PCR products were electrophoresed on a 1.6% agarose gel with a 1000-bp DNA size marker at 100 V for 25 min.

### 4.9. Statistical Analysis

All assays were replicated three times for each treatment. We used the websites http://www.ncbi.nlm.nih.gov/ for blast research at the GenBank database. The two-way sequencing results were spliced by DANMAN Version 8 (Lynon Corporation, SanRamon, CA, USA) and used MEGA Version 7.0.26 (The Pennsylvania State University, University Park, PA, USA) to analyze sequence variability, calculate genetic distance, and construct the phylogenetic tree [38]. The evolutionary distances were computed using the Maximum Composite Likelihood method [39] and are in the units of the number of base substitutions per site.

## Figures and Tables

**Table 1:** Mycotoxin contamination in studied medicinal herbs.

Scientific Name	Sample Name	Mean ± SD (μg kg^−1^) *					
		AFB_1_	AFB_2_	AFG_1_	AFG_2_	OTA	CTN
*Notoginseng* radix et rhizoma	02-1	2.10 ± 0.03	-	-	-	-	-
	02-2	1.29 ± 0.02	-	-	-	-	-
	02-3	1.89 ± 0.08	-	-	-	-	-
*Codonopsis* radix	03-1	1.08 ± 0.04	-	-	-	420 ± 5.99	-
	03-2	0.89 ± 0.02	-	-	-	360 ± 3.71	-
	03-3	1.56 ± 0.08	-	-	-	515 ± 9.23	19 ± 0.41
*Scutellariae* radix	04-1	0.24 ± 0.01	-	-	-	49 ± 1.74	-
	04-2	-	-	-	-	178 ± 2.13	53 ± 0.98
	04-3	-	-	-	-	231 ± 3.44	15 ± 0.46
*Morindae officinalis* radix	05-1	0.33 ± 0.01	-	-	-	-	-
	05-2	0.12 ± 0.01	-	-	-	-	-
	05-3	0.14 ± 0.01	-	-	-	-	-
*Ganoderma lucidum*	06-1	2.19 ± 0.03	-	-	-	0.79 ± 0.02	-
	06-2	1.80 ± 0.12	-	-	-	1.84 ± 0.02	-
	06-3	3.76 ± 0.16	0.43 ± 0.003	-	2.11 ± 0.02	0.48 ± 0.03	-
	06-4	1.81 ± 0.07	0.50 ± 0.02	-	0.87 ± 0.02	0.35 ± 0.01	-
	06-5	1.12 ± 0.02	-	-	-	-	-
	06-6	0.94 ± 0.04	-	-	-	-	-
	06-7	1.16 ± 0.04	-	-	-	-	-
*Coicis* semen	07-1	1.71 ± 0.04	-	-	-	-	-
	07-2	0.28 ± 0.01	-	-	-	-	-
	07-3	0.21 ± 0.01	-	-	-	0.81 ± 0.01	-
*Amomi* fructus	08-2	-	-	-	-	11.4 ± 0.28	-
*Tremella fuciformis*	10-1	0.72 ± 0.02	-	-	-	-	-
	10-2	0.80 ± 0.02	-	-	-	-	-
	10-3	3.05 ± 0.09	-	-	-	-	37 ± 0.77
	10-4	1.87 ± 0.05	-	-	-	-	-
	10-5	2.59 ± 0.08	-	-	-	-	-
*Lentinus edodes*	11-1	0.66 ± 0.03	-	-	-	-	-
	11-2	0.31 ± 0.02	-	-	-	-	-
	11-3	0.25 ± 0.02	-	-	-	-	-
*Poria*	12-1	0.74 ± 0.02	-	-	-	-	-
	12-2	0.56 ± 0.03	-	-	-	-	-
	12-3	0.70 ± 0.01	-	-	-	-	-
	12-4	0.62 ± 0.01	-	-	-	-	-
	12-5	0.51 ± 0.02	-	-	-	-	-
	12-6	0.50 ± 0.01	-	0.85 ± 0.02	-	-	-
*Lycii* fructus	13-1	-	-	-	-	1.84 ± 0.09	-
	13-2	-	-	-	-	0.46 ± 0.02	-

“-” below the limit of detection (LOD). Nine samples that were negative for mycotoxins below the LODs have not been shown in Table 1. The samples were *Jujubae* fructus (01-1; 01-2; 01-3), *Amomi* fructus (08-1; 08-3), *Polygoni multiflori* radix (09-1; 09-2; 09-3), and *Lycii* fructus (13-3). “*” the mean of mycotoxins’ production and standard deviation (SD).

**Table 2:** Primer sets for genes involved in aflatoxin biosynthesis.

Mycotoxins	Gene Target	Primer	Sequence (5’–3’)	Amplification Product (bp)	Reference
Aflatoxins	*aflR*	AflR F AflR-R	CGAAAGCTCCGGGATAGCTGTACG CCGTCAGACAGCCACTGGACACGG	979	[20]
	*omt-1*	Omt F Omt R	GTGGACGGACCTAGTCCGACATCAC GTCGGCGCCACGCACTGGGTTGGGG	797	[20]
	*nor1*	Nor F Nor R	ACCGCTACGCCGGCGCTCTCGGCAC GTTGGCCGCCAGCTTCGACACTCCG	397	[21]
	*ver-1*	Ver F Ver R	GCCGCAGGCCGCGGAGAAAGGTGGT CCGCAGTCAATGGCCATGCAGCG	452	[20]

**Table 3:** Thirteen different species of medicinal herbs from China.

Scientific Name	No. of Samples	Sample Name	Producing Regions
*Jujubae* fructus	3	01-1; 01-2; 01-3	Hebei
*Notoginseng* radix et rhizoma	3	02-1; 02-2; 02-3	Yunnan
*Codonopsis* radix	3	03-1; 03-2; 03-3	Gansu
*Scutellariae* radix	3	04-1; 04-2; 04-3	Gansu
*Morindae officinalis* radix	3	05-1; 05-2; 05-3	Guangdong
*Ganoderma lucidum*	7	06-1; 06-2; 06-3; 06-4; 06-5; 06-6; 06-7	Shanxi
*Coicis* semen	3	07-1; 07-2; 07-3	Fujian
*Amomi* fructus	3	08-1; 08-2; 08-3	Yunnan
*Polygoni multiflori* radix	3	09-1; 09-2; 09-3	Guizhou
*Tremella fuciformis*	5	10-1; 10-2; 10-3; 10-4; 10-5	Fujian
*Lentinus edodes*	3	11-1; 11-3	Hubei
		11-2;	Zhejiang
*Poria*	6	12-1; 12-2; 12-3; 12-4; 12-5; 12-6	Anhui
*Lycii* fructus	3	13-1; 13-2; 13-3	Xinjiang

## Supplementary Materials

Supplementary materials can be accessed at: https://www.mdpi.com/2072-6651/12/1/30/s1, Figure S1: HPLC-FLD chromatograms for (**A**) AFB_1_, AFB_2_, AFG_1_, AFG_2_ standard (AFB_1_, AFG_1_ = 10 ng mL^−1^; AFB_2_, AFG_2_ = 3 ng mL^−1^); (**B**) AFB_1_, AFB_2_, AFG_2_ positive samples (*Ganderma lucidum* 06-3); (**C**) OTA standard (OTA = 20 ng mL^−1^); (**D**) OTA positive samples (*Codonopsis* radix 03-1); (**E**) CTN standard (CTN = 10 ng mL^−1^); (**F**) CTN positive samples (*Tremella fuciformis* 10-3). Figure S2. HPLC-MS/MS chromatograms with MRM modes for (**A**) AFB_1_, AFB_2_, AFG_1_, AFG_2_ standard; (**B**) OTA standard; (**C**) CTN standard; (**D**) AFB_1_, AFB_2_ positive strain (*Aspergillus flavus* isolated from *Amomi fructus* 08-3); (**E**) CTN positive strain (*Aspergillus chevalieri* isolated from *Amomi fructus* 08-1). Two precursor-to-product ion transitions were simultaneously monitored at *m*/*z* 313.0–285.0, *m*/*z* 313.0–269.0 for AFB_1_; *m*/*z* 315.0–287.1, *m*/*z* 315.0–259.1 for AFB_2_; *m*/*z* 329.1–243.2, *m*/*z* 329.1–311.0 for AFG_1_; *m*/*z* 331.0–313.0, *m*/*z* 331.0–245.0 for AFG_2_; *m*/*z* 404.1–239.0, *m*/*z* 404.1–358.0 for OTA; and *m*/*z* 251.0–233.0, *m*/*z* 251.0–205.0 for CTN. Table S1: Calibration curve, limit of quantification and limit of detection of six mycotoxins.
