# Supplementary material for: Occurrence and Characterization of Fungi and Mycotoxins in Contaminated Medicinal Herbs
Source: Toxins (Basel). 2020 Jan 3;12(1):30. doi: 10.3390/toxins12010030 (PMC7020482; doi:10.3390/toxins12010030)
Supplement: Supplementary file 1 [file toxins-12-00030-s001.pdf]

Supplementary Materials: Occurrence and Characterization of Fungi and Mycotoxins in Contaminated Medicinal Herbs

Ling Chen, Weipeng Guo, Yuqing Zheng, Jinzhen Zhou, Tingting Liu, Wei Chen, Daqing Liang, Meiping Zhao, Yudan Zhu, Qingping Wu and Jumei Zhang *

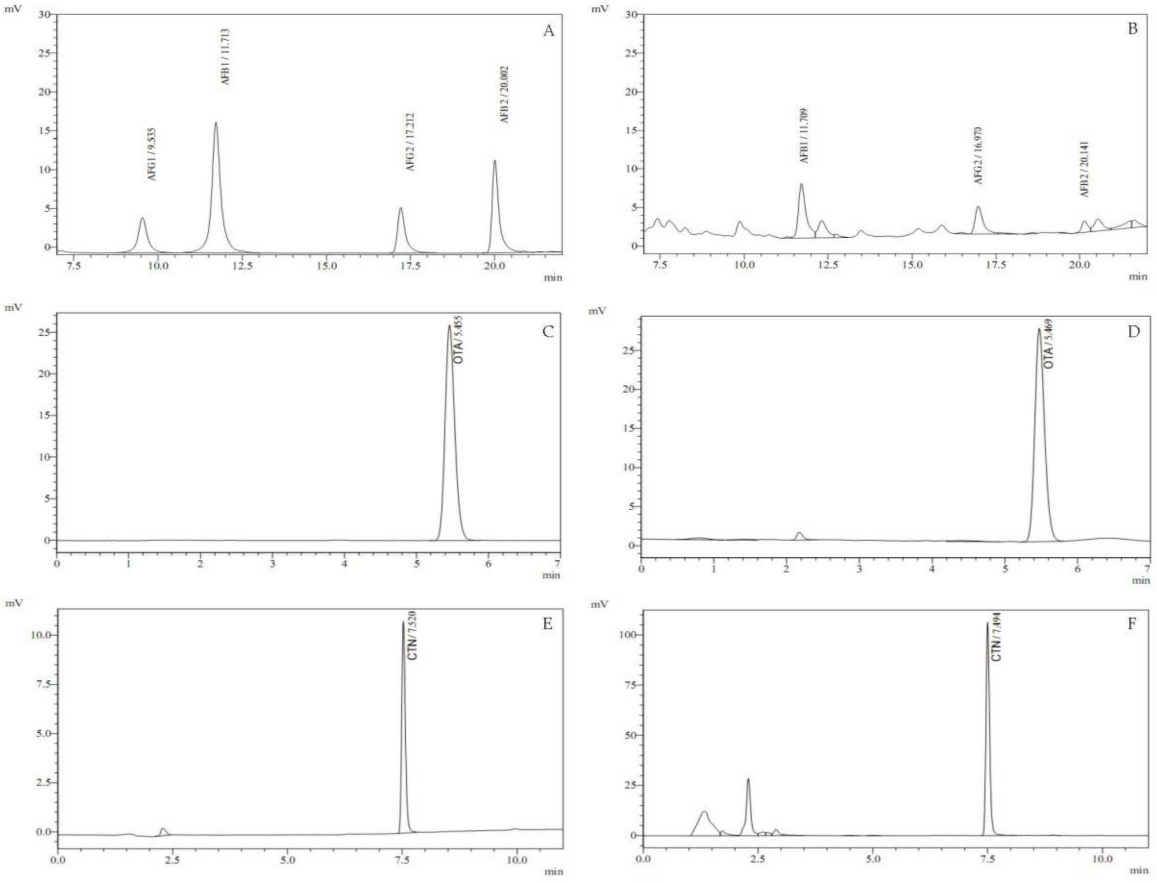

Figure S1. HPLC-FLD chromatograms for (**A**) AFB_1, AFB_2, AFG_1, AFG_2 standard (AFB_1, AFG_1 = 10 ng mL^{-1}; AFB_2, AFG_2 = 3 ng mL^{-1}); (**B**) AFB_1, AFB_2, AFG_2 positive samples (*Ganderma lucidum* 06-3); (**C**) OTA standard (OTA = 20 ng mL^{-1}); (**D**) OTA positive samples (*Codonopsis* radix 03-1); (**E**) CTN standard (CTN = 10 ng mL^{-1}); (**F**) CTN positive samples (*Tremella fuciformis* 10-3).

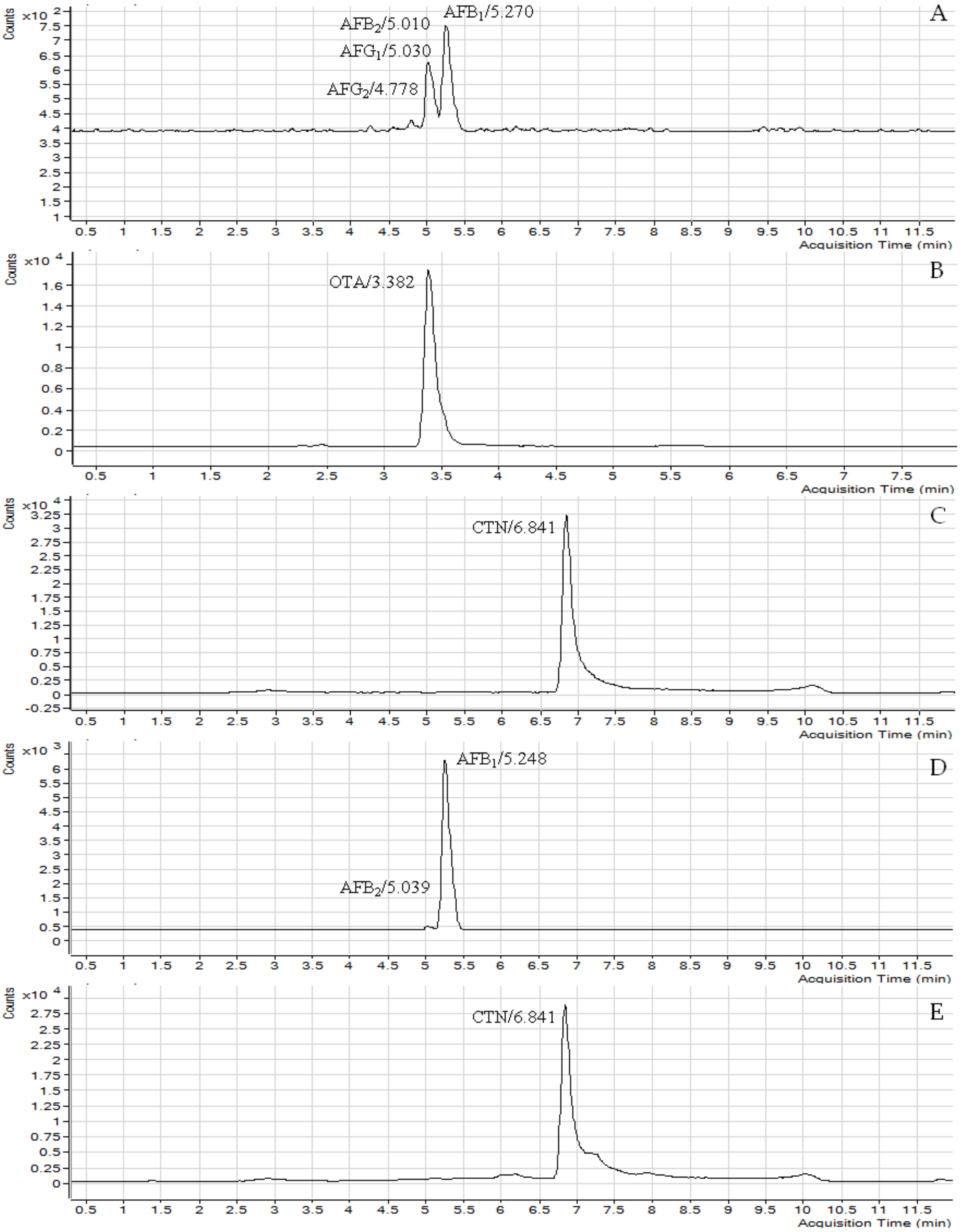

Figure S2. HPLC-MS/MS chromatograms with MRM modes for (**A**) AFB_1, AFB_2, AFG_1, AFG_2 standard; (**B**) OTA standard; (**C**) CTN standard; (**D**) AFB_1, AFB_2 positive strain (*Aspergillus flavus* isolated from *Amomi* fructus 08-3); (**E**) CTN positive strain(*Aspergillus chevalieri* isolated from *Amomi* fructus 08-1).Two precursor-to-product ion transitions were simultaneously monitored at *m*/*z* 313.0–285.0, *m*/*z* 313.0–269.0 for AFB_1; *m*/*z* 315.0–287.1, *m*/*z* 315.0–259.1 for AFB_2; *m*/*z* 329.1–243.2, *m*/*z* 329.1–311.0 for AFG_1; *m*/*z* 331.0–313.0, *m*/*z* 331.0–245.0 for AFG_2; *m*/*z* 404.1–239.0, *m*/*z* 404.1–358.0 for OTA; and *m*/*z* 251.0–233.0, *m*/*z* 251.0–205.0 for CTN.

Table S1. Calibration curve, limit of quantification and limit of detection of six mycotoxins.

Mycotoxin	Linear Equation	Linear Range (ng mL^{-1})	Correlation Coefficient	LOQ (LOD) (μg kg^{-1})	Reference
AFB_1	Y = 59369X − 8373	0.1–50	0.9998	0.042 (0.012)	[28]
AFB_2	Y = 72146X − 2738	0.03–15	0.9998	0.023 (0.0068)	
AFG_1	Y = 24178X − 4037	0.1–50	0.9998	0.10 (0.030)	
AFG_2	Y = 41168X − 1570	0.03–15	0.9998	0.040 (0.012)	
OTA	Y = 7508X − 108	1–50	0.9999	0.12 (0.040)	[29]
CTN	Y = 3002.98X − 33.74	2.5–50	0.9991	4.2 (1.3)	[30]
